# Men's perspectives of caring for a female partner with cancer: A longitudinal narrative study

**DOI:** 10.1111/hsc.13956

**Published:** 2022-08-10

**Authors:** Jenny Young, Austyn Snowden, Richard G. Kyle, Rosie Stenhouse

**Affiliations:** ^1^ Edinburgh Napier University Edinburgh UK; ^2^ University of Exeter Exeter UK; ^3^ University of Edinburgh Edinburgh UK

**Keywords:** cancer, carer, family carer, gender, masculinity, men, narrative

## Abstract

Increasing evidence on men's involvement in informal, unpaid care has not transferred to the research literature around men's *experiences*. The aim was to explore the perspectives of men who are caring for a female partner with cancer over 1 year. Longitudinal narrative interviews (*n* = 22) were conducted with eight men in the UK from 2018 to 2019. Participants were aged from 32 to 76 years old, were all white British and in heterosexual relationships with women diagnosed with a range of cancer types. Interviews were transcribed and then analysed using a structural and performance approach to narrative analysis. We present, across four scenes, a process of change, transition and emotion management as the men were launched into a role that came with new responsibilities and expectations. Our study advances knowledge by highlighting the way that men perform and reflect on their negotiation with masculine discourses while supporting their partner, with implications for policy, research and practice.

**What is known about the topic**

- There is a large field of research on the challenges associated with caring for someone with a cancer diagnosis.
- However, there is a lack of research that considers how men who care may actively construct and present themselves in a particular way and how constructions of masculinity interact with outcomes and experiences.

**What this paper adds**

- Findings highlight the way that managing impressions and presenting particular fronts generated distress when it masked hidden or ‘true’ emotions.
- Caring is not a static concept but something that is constantly evolving. Longitudinal research like this is valuable for gaining insight into the fluctuating emotions and altered perspectives that can remain years after diagnosis.
- It should not be assumed that there is a relationship between someone feeling distressed and their likelihood of taking actions to reduce that distress. If individuals do not see themselves as a ‘carer’, it is likely to impact on their uptake of support.

## 1 INTRODUCTION

Across the world, due to circumstances such as illness, frailty and disability, people provide unpaid assistance and support to their relatives, partners and friends. Within policy and literature this person is usually referred to as a ‘carer’ or ‘caregiver’. In the United Kingdom (UK), one in eight adults (6.5 million people) are classed as carers (Carers UK, 2022). Due to an ageing population, more effective treatments for illnesses such as cancer, a decline in local authority funding and now COVID‐19, the carer population is increasing at a faster rate than population growth (Larkin et al., 2019). Rising demand for informal care, therefore, makes it increasingly important to have a comprehensive understanding of carers' experiences. However, less is known about the experiences of men who care. This limits understanding on how to provide tailored support. Therefore, this study addresses that evidence gap.

### 1.1 Gender and informal care

Within most countries and cultures, the majority of informal care is carried out by women (Verbakel et al., 2017). However, men also provide a substantial amount of care: around 4 in 10 carers are men in the United States (44%) and Scotland (41%), and around half (49%–51%) of all carers in Canada (Baker et al., 2010; National Alliance for Caregiving (NAC), 2020; Scottish Government, 2015). Yet, in the main, increasing evidence on the extent of men's involvement in care has not transferred to the research literature around men's *experiences* of providing care. A systematic review to assess the gender balance of study samples of family carers of someone living with cancer found that across 82 papers published since 1995, 35.5% of participants were men and 64.5% were women (Young et al., 2021).

There is a large field of research on the challenges associated with caring including, psychological distress, feelings of uncertainty, financial strain, unmet needs, changes in sexual relationships and establishing a carer identity (Coelho et al., 2020; Geng et al., 2018; Levesque et al., 2022; Strauss et al., 2019; Taylor et al., 2021; Witham et al., 2018). Yet, male carers' experiences of masculinity and the way that wider social structures and discourses shape these understandings are largely unexplored. Some scholars have concluded that due to higher rates of distress, depression and anxiety women are more emotionally burdened by caring than men (Pinquart & Sörensen, 2006; Swinkels et al., 2019). However, there is a lack of research that considers how gender, as a socially constructed phenomenon, rather than a measurable trait, interacts with outcomes and experiences (Moynihan, 2002). This is an oversight as quantitative (and particularly cross‐sectional) research does not shed light on how men and women who care make sense of their family and caring circumstances. Therefore, *why* caring can be so challenging and, in turn, why there may be differences (and similarities) in experiences between men and women requires attention.

### 1.2 Male caring

There have been some useful insights made in relation to older men's experiences (Calasanti & King, 2007; Milligan & Morbey, 2016; Spendelow et al., 2017; Willis et al., 2020). Within the field of cancer care, the small but growing evidence base on men's experiences has highlighted topics such as: low levels of support (Bigatti et al., 2011), difficulties in expressing and providing emotional support (Lopez et al., 2012; Zahlis & Lewis, 2010), being ‘seen’ and recognised within the healthcare setting (Chen et al., 2004; Hilton et al., 2000; Maughan et al., 2002), wanting to protect but also feel cared for (Oldertrøen Solli et al., 2019), engaging in a task‐oriented approach to caring (Mazanec et al., 2018; Ussher & Sandoval, 2008) and putting the needs of the care receiver before their own (Hilton et al., 2000; Mazanec et al., 2018). Consequently, there are unique features in these experiences relating to approaches to caring, coping style and the expression of emotion. Therefore, research on men's experiences of caring should be methodologically attentive to the nuances and contradictions within a concept such as gender. A constructionist narrative framework meets these requirements here, as it captures identity, experiences, actions and meaning within the social and cultural context in which they occur (Burr, 2006). Taking these ideas forward, using a qualitative narrative approach, the research question is: what is it like to be a male and care for a partner with a diagnosis of cancer over one year?

## 2 RESEARCH METHODS

Ethical approval was obtained by Edinburgh Napier University ethics committee in 2017 (reference: FHLSS/1729). A longitudinal, narrative design was used in this study. Narrative approaches rest on the assumption that storytelling is an inherently human and universal way of expressing experience (Riessman, 2008). Articulating the caring experience from a first‐person perspective, generates a framework in which the speaker can weave together illness experiences into their life. A longitudinal narrative approach is well‐suited to a study about caring within illness as it recognises that, over time, the participant's perspectives and relationship dynamics may stabilise and/or change and their emotional and behavioural responses may fluctuate in multiple and contradictory ways (Saldaña, 2003; van der Kamp et al., 2021). In summary, our study sits within a social constructionist ontology and so we chose a methodology that was congruent with this position. Narrative interviews allowed us to interpret the identities, perceptions and relationships of the participants, within a caring context.

### 2.1 Recruitment

The target study population were adult men who resided in Scotland who were currently supporting their partner/spouse due to a diagnosis of cancer. There were no exclusions based on relationship type (married, cohabiting, living apart), length of time in the relationship or cancer type. Time from diagnosis was set from 3 to 12 months (at the first interview) to capture current caring experiences, including, for example, adjustment to their partner's illness and significant clinical moments such as their partner receiving treatment.

Participants were recruited through three Scottish cancer organisations who either posted out a letter of invitation or added a notification about the study on their social media accounts (i.e. Facebook and Twitter). Postal letters contained a participant information sheet, a reply slip and a stamped addressed envelope (addressed to the lead researcher). Eighty letters were posted and four participants were recruited through this method. Two participants were recruited through social media. One further participant was recruited through word of mouth after placing a recruitment poster in the lead researcher's place of work. This recruitment strategy spanned 14 months and so the decision was made, after the eighth participant was recruited (21 interviews conducted at that point), that this was a sufficient sample size. This decision was based on both practical considerations (time remaining to finish the study) and as data collection and analysis had been running in tandem with each other, our acquired depth of understanding on the relationship between masculinity and caring (Hennink et al., 2017).

### 2.2 Sample characteristics

A total of eight men aged from 32 to 76 years participated in this study. All the men were in heterosexual relationships. Seven were married and one couple were cohabiting. Five were in employment and three were retired. Cancer types included: breast, oesophageal, cervical and skin. Types of treatment included chemotherapy and surgery. Average time from diagnosis at interview one was 10 months (range 7 months to 1 year). All names have been replaced with pseudonyms to provide anonymity (Table 1).

**TABLE 1:** Participant characteristics

Participant	Age	Partner's primary cancer diagnosis	Time from diagnosis at interview one	Relationship status
Mark	50	Breast	One year	Married for 15 years
Stuart	72	Oesophageal	8 months	Married for 50 years
Jack	32	Cervical	7 months	In a relationship for 14 years
David	58	Breast	1 year	Married for 10 years
Brian	62	Breast	8 months	Married for 11 years
Paul	50	Breast	1 year 2 months	Married for 10 years
James	76	Skin	1 year	Married 20 years
Angus	71	Breast	1 year	Married 20 years

### 2.3 Data collection

Each participant was invited to take part in an interview in a location, and a day and time, of their choice. Most (*n* = 5) wanted to meet in their home. Other settings included a café, a library, a private room in a charity support centre and a private room in a cancer hospital. Written consent was obtained from every participant before their first interview and verbal consent was taken for subsequent interviews. Interviews were conducted face to face from 2018 to 2019.

First interviews began with the researcher stating “I would like you to tell me your story of what it is like to care for your partner who has a cancer diagnosis”. A short topic guide was developed that covered the following broad areas: caring roles and responsibilities, relationship dynamics (and any changes), social/practical challenges and support needs. However, the overall aim was to allow the participants to speak freely and to have control over the direction of the interview (McAdams, 1993).

Seven participants were interviewed three times over 1 year and one participant once (22 interviews in total). This was because one participant withdrew from the study after his first interview. Following each interview participants were debriefed, which involved asking how they felt and asking if they would like information on how to access support from a cancer charity. All participants declined the offer of further support and most then thanked the researcher for being given the opportunity to talk. Single interviews lasted from 32 to 74 minutes. After each interview the lead researcher emailed a typed account of the interview to the participant and asked if they felt it was an accurate summary of the conversation; all were in agreement that it was.

### 2.4 Data analysis

To enable a multidimensional interrogation of the male caring experience, the analytic framework layered two approaches: structural and performative. First, narrative segments were identified as guided by Labov and Waletzky's (1997) seminal six‐part structural model. Next, performative approaches recognise that stories are not told in vacuums they are told to someone (Riessman, 2008). This stage of the analysis focused on the participant's positioning and the wider context including the researcher's influence, the setting and the social circumstances. To help facilitate this process, we worked through a series of questions and considerations, drawing on the guidance of other scholars, as noted in Table 2. The team of four researchers reviewed three transcripts independently then came together to discuss their interpretations. Author one then continued to develop the analysis using a holistic approach. In holistic approaches, an individual's story is viewed as a whole (Lieblich et al., 1998). This helped to keep the participants stories intact in order to preserve the caring ‘journey’.

**TABLE 2:** Analytical questions

Positioning	How do narrators position themselves? (Bamberg, 2012) How do I (interviewer) position myself to the participant and how do I respond emotionally and intellectually? (McCormack, 2004)
Characterisation	How do participants construct characters and define (or not) their relationship to the character?
Co‐construction	How are stories co‐constructed?
Context	How does the setting influence the conversation? What sociocultural assumptions/norms influence the participants' storytelling? (Murray, 2000)
Performance	Why was the narrative developed that way, and told in that order? In what kinds of stories does the narrator place himself? How does the participant strategically make preferred identity claims? What other identities are performed or suggested? What was the response of the listener/audience, and how did it influence the development of the narrative and interpretation of it? (Riessman, 2003)
Illness narratives	How does the participant respond to the idea of a ‘disrupted’ experience and what do such narratives reveal about identity experience and culture? (Bury, 2001) How does the participant understand illness? (Charmaz, 1991; Frank, 1995)

This process then formed the basis of longer accounts of analysis which were shared with the research team for feedback and discussion. This was an iterative process that took about 5 months and generated a focus on how participants positioned themselves to the researcher, themselves and others and the unfolding of illness in the experience of someone's life (Bury, 2001).

At this point, the findings were written up on a case by case basis (Beal, 2013). This generated eight individual stories of the male caring experience (Young, 2021). Next (and presented below) commonalities between the cases were written into a series of ‘scenes’, as would be found in a theatrical play. Presenting the findings this way draws attention to the performative nature of identity, the way actions, behaviours and identity positioning can change and fluctuate over time, and they symbolise the men's narratives taking ‘centre stage’. Considering that each scene was an episode, the intention was twofold: to shape the data from four isolated events to a logical sequence with meaning, and to recognise both the individual person and the contribution of wider context. This draws out the connections between participants, motivations and action in order to generate explanations for particular outcomes (Polkinghorne, 1995).

## 3 FINDINGS

These four scenes represent a year spent caring (Table 3). In essence, they reflect change, transition and self‐awareness as the men were launched into a role that came with new responsibilities and expectations.  

**TABLE 3:** The male caring experience in four scenes

Scene	Overview
‘In that moment life changes’	Adjusting to the news of their partner's diagnosis
‘Caring but not a carer’	Recognising and resisting being a carer
‘Opening the valve’	Perceived needs and use of support
‘Repercussions’	The long‐term consequences of caring

### 3.1 Scene One—‘In that moment life changes’

After the opening request to ‘tell me your story’ most of the participants, without hesitation, began to describe the lead up to their partner's diagnosis. Their reactions included shock, fear, denial and anger. However, two of the participants, rather than describing how they felt in the moment or the days following their partner's diagnosis, shifted their perspective to allow them to reflect back:

They said it's cancer and it changed everything completely. (Paul)

They described changes to their daily routines, to their employment and to the dynamics within their relationships. Negative emotions arose when the men began to reflect on the permanency of the change in terms of psychological and physical side effects:

She's had the mastectomy that's a loss. You know she said it's just a lump of fat with a nipple on the end but to a man it's a bit more than that. (David)

For the most part, as change was unplanned and unwanted the men struggled to adjust to their current situation. David summarised the challenges associated with change as ‘loss’. As his quote exemplifies, changes to his wife's physical appearance also affected him. He suggested that there was deeper significance to his wife's breast than it just being a ‘lump of fat’ and he made masculinity relevant to this discussion by stating this view was not just held by him but by ‘men’.

What was particularly challenging for these men was how, in trying to adjust to change, it led to a longing for their previous ‘normal’ life, before cancer. Jack described the feeling as being “derailed” which captured the way that caring had impacted on his impetus to move forward. It was this state of flux of being caught between trying to adjust but hoping for a return to their earlier life that was upsetting:

It has left scars and wounds and normality as we knew it has gone. (David)

David's use of ‘scars and wounds’ had double meaning. His wife was left with physical scars following the mastectomy but these scars also symbolised permanent emotional pain. As the men tried to come to terms with the realisation that their ‘old’ life had gone, they were left with no choice but to move into a new reality.

### 3.2 Scene Two—‘Caring but Not a Carer’

After the initial shock began to fade, this second scene marked the point in which the men began to transition into their new roles. In the main, this was described in terms of their involvement in new tasks and responsibilities. For example, accompanying their partner to hospital visits, taking on a greater share of cooking, shopping and cleaning, administering medical care and providing emotional support.

When the care needs of their partners increased beyond the normal boundaries within their existing relationship, all of the men spoke about altering their behaviour in some way but with varying degrees of struggle. Notably, they were attempting to “do it all” without support from any other services. Their need to take control and provide for their partners, as will be discussed, highlights the social construction of men as essentially autonomous and independent. Paul left his job to provide full‐time care as they had no other sources of support. Other changes included working different hours at work to accommodate their partner's hospital visits and spending much less time on their own hobbies and interests (issues that affected working men and retirees differently). Accordingly, the magnitude and nature of these adjustments had an impact on their self‐identity. Stuart, for example, jokingly referred to himself as an “auxiliary nurse” as he described administering medication and cleaning his wife's feeding tube. Yet, the majority of the participants in this study did not identify with the term carer. The essence of this was captured by Stuart when he stated:

I'm caring but I'm not a carer. (Stuart)

It was notable that across all of the interviews no one referred to a precise or defining moment when they felt they had transitioned into or acquired the role. Moreover, even though they were engaging in tasks that caused them to perceive their role differently, this was done from the position of a husband rather than a carer:

I don't feel like a carer, I feel like a husband (Mark)

In contrast, Jack aspired to feel associated with the role in order to reinforce his sense of self‐worth. However, he questioned the suitability of the term to summarise his contribution as he was not carrying out many ‘physical tasks’. The implication being, individuals may transition into the carer role with pre‐existing expectations around the type of care that they *should* be providing. But when these expectations are not met it can cause further strain.

Not everyone dismissed the term. Paul, immediately after stating he had given up his job, referred to himself as a “full‐time carer”. The juxtaposition of these two pieces of information seemed intentional to highlight how time consuming the role was. Therefore, this justified his decision—he cannot work as caring is a full‐time job.

### 3.3 Scene three—‘Opening the Valve’

There were clear statements of distress from all of the men reflecting the negative emotional impact of caring:

I didn't want her to actually see that I'm a wimp. Because that's what I felt like. I thought why am I not handling this, it was really, really terrifying. (Stuart)

There were expressed needs for more information on the cancer diagnosis, dealing with side effects and assistance with medical equipment. Yet, their distress and information needs rarely prompted any form of action. For the most part, the desire to be strong and stoic and to protect their partner were prioritised over their own needs or well‐being.

Stuart did not perceive any need for support as he did not self‐identify as a carer:

I don't think I'm doing anything special I'm just doing what I'm supposed to be doing in sickness and in health. (Stuart)

However, he later described attending a patient support group and listening to shared experiences helped to normalise his situation and reduce worry. Then by interview three, he was more open to the idea of accessing support. He talked with greater insight into how he should ‘let off steam’ to help prevent stress from building up and that once ‘the valve has been opened’ through talking to others, it helped to reduce stress. Notably, it took Stuart 1 year to make this realisation as until then, his time, focus and energy had been on his wife.

Like Stuart, David went through a process in which the way he viewed his roles and responsibilities, within the context of his relationship, had implications for his perceived need and use of support. For example, when his wife was first diagnosed he emphasised how reliant she was on him. The ‘emotion work’ involved in supporting his wife meant suppressing his own needs and practically, as he was confined to the house, he could not travel to access support. In his own words he felt ‘lonely and isolated’. Consequently, this prompted him to seek local guidance from friends. This step—or to carry on with Stuart's analogy—this ‘opening of the valve’ appeared to change his perceptions on accessing support. However, he was aware of the way in which masculine discourses can position, explain and restrict help seeking behaviours. He used different strategies to explain and justify gendered support utilisation and to generate a masculine position that men do not seek out help but women do. This allowed him to position himself as the exception and he regained strength from being vulnerable:

I've spoken to other husbands and their wives have had cancer and they very much clam up, don't want to talk about it, it's a weakness But men need to. It's having to realise it…your own vulnerability in that. And you can't hit every expectation. Not beat yourself up too much about. (David)

### 3.4 Scene four—‘Repercussions’

In this final scene the participants' stories come to a conclusion. Sadly, for two of the participants, their spouse passed away before the final interview. Yet, for everyone there were no clear endings. Their stories seemed incomplete or ‘in‐process’ as the men were still grappling with the long‐term repercussions of caring. One particular element that was deeply impacted was the spousal relationships. For Mark, Jack, David and Paul changes brought on by their partner's diagnosis caused stress, leading to arguments, distance and moments of tension.

Jack and David both reflected on how, as the distance between them and their partner increased, it led to feelings of apprehension and loneliness:

Very distant with each other. But with each other more than we've ever been, in the full time we've been together. Distant, but together all the time. So, it's just small talk, it's weird. (Jack)

Second and third interviews were held 1–2 years from their partner's diagnosis. However, even after this amount of time, for most of the men, persistent feelings of uncertainty and/or fear of recurrence remained as a pressing concern. Brian and Stuart's partners both showed signs of physical improvement but their health remained a stressor:

The worry never leaves me. And until somebody actually physically tells me, and tells her, this is not coming back, you're alright, I'm still going to have that thought. (Brian)

Feeling constantly worried and “on call” left a strain on the men. Learning to live with these feelings and overcome the uncertainty was going to be an ongoing challenge. There were mixed reactions to how they felt they were going to master a life of uncertainty.

There was a desire to try to establish something meaningful from the uncertainty. This perspective was particularly expressed in final interviews. It seemed some of the men wanted to try and provide a conclusive ending to their stories:

So, I don't know if I'll ever get off this path. I don't think I want to come off it, I think I want to stay on it as the protector (Brian)

Brian found meaning in the ‘journey’. His unfolding realisation highlighted the positive benefits that can be associated with caring. While the men still faced challenges, feeling like they were moving towards something was used to re‐establish a sense of control and emotional adaption. Yet, some participants could not move forward. For the men who were now bereaved they would need time and support to establish new roles and ways of being (Table 4).

**TABLE 4:** Supplementary quotes

Scene	Further Illustrative quote
‘In that moment life changes’	Everyone is living their normal life but you can't and you don't feel jealous towards it, it's just a loneliness. Feeling as you want to step back into how things were before but you can't, as you have just got a weird thing hanging over you. (Jack)
‘Caring but not a carer’	I go to medical appointments, this is your partner, your carer, your carer, your partner, and I don't know if I like the word carer. I know that's what people do – I'm thinking to myself, no, that's my love and I want to make her better. You know, it's just like children really, isn't it, you go out of your way to do anything for them.’ (Paul)
‘Opening the valve’	So, I do not like these things [support groups], my name's X and I'm an alcoholic, I just feel very uncomfortable. But anyway, I went in and it was brilliant. To hear other people talking and realising they are not exclusive to you. But when you hear someone else who has had the same operation and is suffering in a very similar way, I'm thinking, alright, so I don't need to unduly worry.’ (Stuart)
‘Repercussions’	And you are always going to have regret because you are walking on a road you have never walked before. And if you have a chance to do it a second time, you will do things differently. (Angus)

## 4 DISCUSSION

To our knowledge, this is the first, longitudinal, narrative study to explore the experience of caring for a partner with cancer from the perspective of men. The exploration of what factors shape the enactment of gender—the socially guided and interactional activities that are expressed as masculine gendered identity—are largely absent from the cancer carer literature.

As captured in scene one, change was a significant part of the men's experiences. The magnitude behind this change was considerable—‘life’ and everything that had been known and taken for granted had gone. Therefore, the men engaged with different strategies while performing a number of roles including the protector, the provider and the stoic man who shielded his pain from his family in order to adapt.

One way to explore masculinity, in this sample of men, was to consider the way that it socialised them to adhere to particular masculine norms (Kenny et al., 2020; Spendelow et al., 2017). As discussed in scenes two and three, the men rarely disclosed their feelings of strain to anyone and crucially not to their partner. They spoke about ‘just getting on with it’, were reluctant to cry in front of others and blocked negative emotions such as worry and fear in order to project a calm and resilient front. Milligan and Morbey (2016) also found, in their qualitative study, that the men were reluctant or refused to seek help. Their need to take control and provide for their wives meant that seeking support was perceived as failure.

### 4.1 Theoretical contribution

A reoccurring thread across the men's narratives was their eagerness to *protect* their partner, as has been reported in other carer studies (Boele et al., 2017; Oldertrøen Solli et al., 2019). In general, the protector ‘cultural script’ involves men in heterosexual relationships taking a more central and protective role than women (Glick & Fiske, 2001). The spousal relationship is a key context for establishing such a dynamic. However, there was a sense among the men in this study, that in order to cope with the threat to their partner's life this role was brought into greater focus. The men wanted to feel that they had an essential role, particularly within the context a disease such as cancer, which left them feeling fearful and unsettled due to its unpredictable trajectory. Therefore, the positioning into this role may have been fuelled by existential threat rather than the discursive need to be the ‘strong’ one in the relationship. In essence, to counter the threat the men appeared to try and establish meaning and purpose in other ways.

It was also common for the men to talk about their wish to protect their partners from their *own* distress. By concealing their emotions they were fulfilling expected norms that position men as being emotionally closed and invulnerable (Fee et al., 2020). However, this was not that the men had difficulty expressing their emotions, as some scholars have suggested is characteristic of male carers (Li et al., 2013). Indeed, most of the men were very open and candid about their emotions within the interview setting. This was a conscious controlling and suppression of emotions to particular people and within particular contexts. The essence of which was captured by one of the participants when he remarked he was ‘strong enough not to let anyone see he was cracking’. In presenting this image, he was able to reframe his vulnerability as a strength. This may be adaptive in the short term, as it reinforced his positioning as a man who is competently engaging in mastery and coping. However, managing impressions and presenting particular ‘fronts’ (Goffman, 1959) can generate a sense of fragmentation when it masks hidden or ‘true’ emotions.

Consequently, maintaining their protector role required a degree of emotion management. There has been little discussion of this in the carer literature beyond the idea that this may be because of men ascribing to hegemonic values (Hilton et al., 2000). Emotion work theory is underpinned by feminist theory as it shines a light on the often underappreciated and hidden elements of ‘work’ (in the public and private sphere) which are usually done by women (Hochschild, 1985). Therefore, interpreting emotion management within the context of male caring is novel. It encourages reflection on the hidden elements of ‘work’ associated with the role.

### 4.2 Implications for policy, research and practice

It is important to recognise from the outset, there is a risk in referring to ‘men’ as one homogenous group. Consequently, Gough (2018) suggests that intersectionality should act as a reminder not to homogenise groups such as ‘white men’. When considering implications, such as the need to engage with and identify carers (whether male or female), it is acknowledged that every ‘group’ contains a broad array of diversity with numerous connections between characteristics such as gender, race, age, sociodemographic status and so on. Within policy and clinical guidelines (National Cancer Institute, 2019; National Institute Clinical Excellence (NICE), 2020; Scottish Government, 2015) carers are largely conceptualised as one group. As such, the risk is that current and future guidelines on how to support carers will fail, or will only be applicable to certain pockets of the carer population.

Non‐disclosure and the failure to divulge distress to others is recognised to be more common in men than women (Charteris‐Black & Seale, 2013). As discussed in scene two and three, (and wider literature, e.g., Greenwood & Smith, 2015) none of the men, to begin with, accessed any form of formal support. It should not be assumed that there is a relationship between someone feeling distressed and their likelihood of taking actions to reduce the distress. Particularly, as raised in scene two and by other scholars (Molyneaux et al., 2011) if individuals do not see themselves as a ‘carer’ and only wish to be defined by the pre‐existing relationship they are in, it is likely to impact on their uptake of support. Related to this, distress can come in many guises (Balfe et al., 2016) so ‘lower’ levels of distress (predominantly captured in quantitative research, e.g., Perz et al., 2011) in male carers may not mean their concerns or needs feel less severe to them.

Building on this and considering the dynamics of the interview process—when the research topic is deemed to be ‘sensitive’ the interviewer's female gender (as was the case in this study) has been framed as a beneficial resource as it can encourage openness from male participants (Lohan, 2000). Her presence may have enabled different versions of the interviewee's masculine self to be revealed and brought into tension. Other authors point in a different and more complex direction, beyond gender, identifying a set of conditions that may contribute to an ethical and successful interview and data production. As such, Delaunay et al. (2020) note that researching sensitive topics, requires a researcher that can engage in emotional reflexivity in order to build the necessary rapport with participants in the study and show empathy. Gender interacts with factors such as class, age, the psychological state of the participant, the research environment and the sociocultural context of the interview topic (Broom et al., 2009). Therefore, while male and female experiences can be ‘gendered’, no two individual experiences will be the same (Reinharz & Chase, 2002).

### 4.3 Study limitations

Limitations to these claims are acknowledged. From the outset, there was no expectation that these findings would be generalisable. The aim of this research was to study a specific issue within a particular context (Carminati, 2018). In terms of the sample, we prioritised recruiting spousal carers because they usually provide the most intense level of care with a subsequently detrimental impact on their well‐being (Jeong et al., 2020). However, in terms of transferability and representation of the study findings (Guba & Lincoln, 1994), different relationships will have particular dynamics, with consequences for how the role is experienced. Other factors such as proximity to the care receiver (co‐habiting or not) and if the person has other caring responsibilities (such as children and/or elderly parents) will shape perceptions and behaviours. We did not recruit any men who were in same sex relationships, from an ethnic minority group or with a disability (to our knowledge). However, there was no effort to target men from non‐dominant groups (in terms of sexuality and ethnicity). And so, while we did not set out to explore the experiences of caring among more diverse groups, it is recognised that if researchers do not consider a variety of approaches to seek out the voices of those who may not respond to the more obvious forms of recruitment, then the cycle of their underrepresentation in research will continue. However, as recruitment was challenging we used a number of different recruitment strategies, including snowballing, social media and a postal letter to the participant's partner to reach a wider pool of potential participants. These different strategies will have reached different groups of people. For instance, snowballing has the potential to reach ‘hidden’ carers who may not engage with information posted online by cancer charities. This is also an important issue to consider in relation to the point that some people do not relate to or dislike the term carer.

## 5 CONCLUSION

This research advances knowledge by highlighting the way that male carers perform and reflect on their negotiation with masculine discourses while supporting their partner. Our findings illustrate that ‘caring’ is more than the practice of looking after someone. The shift from being a partner, to being someone who provides care because of illness is bound up with different values, assumptions and expectations. Caring is not a static concept but something that is constantly evolving. For that reason, longitudinal research like this is valuable for gaining insight into the fluctuating emotions, altered perspectives, changing relationship dynamics, new responsibilities and the way that life and the caring role can remain closely entwined for years after diagnosis. The two broad and interrelated areas for future work are related to the issues of carer identification and engagement, particularly among ‘hidden’ groups of carers such as men. In essence, our view is that the multifaceted and far‐reaching impact of caring must be viewed through a gendered lens.

## CONFLICT OF INTEREST

All authors declare they have no conflicts of interest.

## Data Availability

Data available on request from the authors.
